# Extra Supplement—The Nursing Mirror

**Published:** 1889-08-31

**Authors:** 


Extra Supplement

### The Nursing Mirror.

All Contributions for this Supplement should be addressed to the Editor, The Hospital, 139-140, Salisbury Court, Fleet Street, London, E.C., and should have the word "Nursing" plainly written in left-hand top corner of the envelope

### En Passant.

SHORT ITEMS.—"E. D.", who writes the "Recollections of a Nurse," is said to be Sister Emma, who nursed Lord Tennyson so skilfully through his illness.—The first party of the new African Mission, of which the Rev. Andrew Murray is president, have sailed for the Cape, but Miss Walton, the nurse, will not join until later in the year.—Miss Elliott, matron-nurse, came in for much praise on the occasion of the opening of the new Cottage Hospital at Ashburton.

HOME NURSING.—Adelaide Ross writes a sensible article in this month's *Quiver* on "Home Nursing." She specially refers to the work of the East London District Nursing Society, which carries into the poorest homes principles of order and cleanliness, and teaches the poor how to nurse themselves. A correspondent lately asked us about ladies of title who have taken to nursing; amongst the "assistant ladies" who visit with the nurses of this society are Lady George Campbell, the Countess of Aberdeen, Lady Greville, and Mdlle. la Baronne Henriette d'Ablaing.

ASYLUM ATTENDANTS.—We are glad to see in the report of the Perth District Asylum that Dr. Campbell has a good word to say for the attendants and nurses. He writes: "There are many good attendants on the staff at present. When it is considered that a good attendant must be healthy of body and healthy of mind, intelligent and active, able to control himself with firmness and others with kindness; that he must be truthful, honest, civil, and submissive to discipline; and when it is considered that his duties are confining, and often irksome and trying, it must be admitted that a man or woman, of whom it can be truthfully said that he or she is a good attendant, has thereby extensive testimony borne to his or her general excellence as a member of society. This class receives less appreciation from the public than it deserves." Miss Gordon, late chief attendant on the female side, has been appointed assistant matron in Miss Robinson's place. Miss Agnes Stewart still acts as matron.

A NURSING GUILD.—In connection with the St. John's Ambulance Association, there exists at Richmond a most excellent nursing guild, of which Miss Murray is secretary. The objects of the guild are: 1. To afford skilful nursing to the poor in their own homes during severe illness, or in case of accident. 2. To form a store of nursing materials (some of which would be for loan only), such as lint, bandages, old linen, linseed meal, turpentine, cotton wool, guttapercha tissue, bronchitis-kettles, water-beds, air-pillows, inhalers, blankets, sheets, nightdresses, waterproof and other materials not generally within the reach of the sick poor, and which medical men are therefore unable to order, however needful they may be. 3. To obtain the services of ladies holding certificates of efficiency for the work, and willing conscientiously to carry out the directions of the doctor in charge of the case. The duties of the associates are: 1. To carry out conscientiously the directions of the medical man. 2. To see the sick person daily, or as often as necessary. 3. To attend to the general sanitation and cleanliness of the sick-room; to apply (when required) poultices, fomentations, etc., etc.; to see to the washing of the patient, changing linen and sheets, dressing wounds, bed sores, etc.; to take temperature and note it on a chart for the inspection of the doctor at his visit, and to procure such nursing material as the case may demand. Similar guilds exist at Leicester and elsewhere, and in those places where there are no district nurses they are of considerable use.

HOMES WANTED.—Will our correspondents who send queries for homes for aged or invalid persons, please give an address to which answers can be sent? We always get so many replies to these queries that we cannot undertake to forward them after this month. Our correspondents would also save themselves some trouble if they worded their queries more precisely. "A small sum" differs in size in the opinion of different persons; it would be better to say £20 or £30, as the case may be.

DOLL COMPETITION.—A nurse kindly informs us that miniature chatelaines for dolls can be had from the firm of Woods, York, price 5s. 3d. Still, we think a little ingenuity would construct cheaper chatelaines, which would look quite as well. Another nurse asks what is to become of the dolls. They will all form an exhibition, which will be free to nurses. The prize dolls will remain the property of the Editor. A country nurse hopes the dolls will go on tour through the country. If they are numerous enough to make an interesting exhibition, we will try and arrange to send them round the provinces. Another nurse writes: "If you knew how busy my life is, and how like an overdone cab-horse I feel, you would not ask me to dress dolls." We do know the perpetual work, the constant weariness of a nurse's life; but sometimes there is a pause. A private nurse, with a small semi-convalescent patient, might amuse both herself and her patient by dressing dolls. A district nurse, sitting up all night, might keep herself awake with this harmless pastime.

TO MEMBERS OF THE BRITISH NURSES' ASSOCIATION.—The following advertisement appeared in last week's *Lancet*: "Hospital Sister wanted. Must be an educated lady, over thirty years of age. A thoroughly trained nurse of considerable experience, and not a member of the British Nurses' Association. Applications, with testimonials and photo, to Z. Y., the *Lancet* Office, 423, Strand, W.C." Similar advertisements have reached our office, and our readers must have noticed the "no member of the British Nurses' Association need apply," which is frequently appended to advertisements. A lady of great experience in nursing, of pleasing appearance and charming manners, has twice lately been within an ace of securing an excellent post as matron, when her prominent connection with the British Nurses' Association has told against her. Until this association weeds out its ranks and adopts a more conciliatory policy, to belong to it cannot be regarded as an honour by our best trained nurses; and, as the above advertisement shows, membership may prove a bar to progress in the profession.

SUMMER AFTERNOONS.—We hope the summer is not yet quite gone, for there are many pleasant little excursions yet to be recommended to nurses. First, there is Epping Forest, which is about the shortest countrified excursion we know. Train to Chingford from Liverpool-street, return fare, third class, 1s. 1d. For river scenery Hampton Court offers many advantages; the picture galleries and the gardens of the old Court are worth seeing, and it is a pretty walk down the river side to Kingston, whence train can be taken home. The return fare from Waterloo, second class, is 2s. The cheapest and quickest way of getting a breath of sea-air is to train to Southend from Fenchurch-street, return fare 2s. 6d. By boat to Southend from Old Swan Pier, London Bridge, takes all day there and back, but is very pleasant, and only costs 3s. We also confess to having enjoyed such a cockney outing as riding outside a 'bus from Piccadilly-circus to the Bull and Bush, Hampstead, and to having enjoyed a frugal tea there on the Heath. As long as coaches are so dear (10s. to Bushey and back) many nurses will have to find pleasure in driving outside the homely 'bus.

### How to Become a Nurse.

"PLEASE, Mr. Editor, what must I do to become a nurse?" Some such query reaches us weekly, and we therefore propose to devote a page to those numerous readers of ours whose nursing experience is yet to come. First of all, let those who wish to become nurses be sure they are in earnest in their wish, and not merely actuated by unwholesome curiosity, weariness of home life, or love of adventure. To tend the sick and dying calls for all that is noblest and best in woman's nature, and a frivolous girl in a hospital ward is a sight to disgust any man. It is scarcely comprehensible how girls can be so unfeeling, so heartless, and so shameless, as to take their society follies and fringes into our great temples of suffering. A building which is ever shaded by the wings of the Angel of Death should be so far hallowed that none should enter it in a spirit of inquisitiveness or frivolity. Let no woman try to become a nurse whose desire is not founded on sympathy with suffering.

But even those who approach nursing work in a proper spirit are not always fitted for the hard work and trying sights of a hospital ward. Therefore, if a would-be nurse can afford it, she had better try her mettle by entering a hospital as a paying probationer for a short period. The usual payment is a guinea a week, and for this sum a probationer is received for not less than three months at Guy's Hospital, Borough; the London Hospital, Whitechapel; King's College Hospital, Strand; and others. County hospitals also take paying probationers; so an applicant who cannot be received at a London hospital is advised to apply to Addenbrookes Hospital, Cambridge, the County Hospital, Brighton, and others. The work differs in the different hospitals; at Guy's and Middlesex the paying probationers are not supposed to do menial work; at St. Bartholmew's and the London no difference is made between the paying and paid probationers. The accommodation for nurses at St. Thomas's, Middlesex, and the London is excellent; but at St. Bartholomew's and Guy's it is not so good. At St. Mary's Hospital, Paddington, special probationers pay £30 a year, and £20 more secures them a bedroom to themselves. Our would-be nurse, then, must consider if she desires to rough it, or to do as little work as possible, before she decides on what hospital she wishes to join.

Another important question is that of the certificate. Without a certificate a nurse is apt to go to the wall, and different certificates have different values. At Middlesex and Guy's paying probationers receive their certificates at the end of a year; at the London and St. Mary's no certificate is given under two years; at St. Bartholomew's, Guy's, and Middlesex, non-paying probationers have to serve three years for their certificate. At Addenbrookes, at Norwich Hospital, and elsewhere in the country, a certificate is given at the end of a year. The second question the would-be nurse has to settle then, is how long she wishes to serve for her certificate.

The nurse who has her living to earn, and cannot begin hospital life gently as a paying probationer, is generally paid £12 the first year, and £18 the second and third years. This is the payment at Guy's. At Edinburgh Royal Infirmary, Newcastle Infirmary, etc., the payment ranges from £20 to £27 after the first year; so it does also at the London Hospital. A would-be nurse had better not hope to earn much while she is being trained, but consider the questions of comfort and certificates first.

Having decided which hospital is best suited to your purpose, then write a letter to the matron and inform her of your wish to become a probationer, and she will return you a series of papers detailing the duties you may expect, and asking questions as to your health, age, former occupation, etc. As a rule, probationers are not taken unless they are tall and strong, and over twenty-four years of age; but exceptions are made in the case of paying probationers. At least two testimonials from professional men as to your character will be required. Probably if these are satisfactory the matron will ask you to call and see her, and if the interview goes off all right, you will retire with orders to wait at home till a vacancy occurs. If you are trying to get into St. Bartholomew's or the London, you may have to wait twelve or eighteen months, whereas you can generally be received at once in the provincial hospitals. During the period of waiting the would-be nurse will have her uniform to make, and she had better study some sensible book on nursing. Domville's "Manual," price 2s. 6d., or Lückes' "Lectures on Nursing," price 2s. 6d., would do; or the matron of the hospital would advise which book she prefers her nurses to read. Attendance at ambulance lectures is also advisable before entering a hospital, if time and opportunity permit.

We have now touched on the principal questions which agitate the would-be nurse; but it has been impossible to give full particulars of the payment, work, etc., of every hospital. We must once more impress upon our many correspondents that the simplest way to gain exact information is to call on the matron of the nearest hospital. To our country friends we recommend this course more than to our London ones, for the matrons of large town hospitals are often so busy they cannot see callers save by appointment. There is no reason why so many would-be nurses should rush to London; the hospitals of Liverpool, Birmingham, and Manchester offer quite as good training as any to be had in the metropolis. Not that we would advise an ambitious nurse to enter a small hospital. Unless there are a hundred beds, and good lectures from the medical staff, the training will be second-rate. Ladies who are only taking up nursing for a short time had better apply to children's hospitals or cottage hospitals, then they will not be kept waiting long for vacancies. Her Majesty's Hospital for Sick Children, Stepney, or Bristol Hospital for Women and Children, or Glasgow Hospital for Sick Children, offer good training in their special branch; and Miss Heanley, matron of Boston Cottage Hospital, gives excellent training to probationers in the work of cottage hospital nurses. The would-be nurse has only got to take *The Hospital Annual*, and there she will find a list of hundreds of hospitals in the United Kingdom, and the number of beds, nurses, etc., in each. Let her choose a large hospital, well-officered, and situated in a healthy locality, and then write to the matron for further particulars.

### LIFE.

Oh! who would live, if only just to breathe
This idle air, and indolently run,
Day after day, the still returning round
Of life's low offices and sickly joys?
But in the service of mankind to be
A guardian good below, still to employ
The mind's brave ardour in heroic aims,
Such as may raise us o'er the grovelling herd,
And make us shine for ever—that is life.

THOMPSON.

### JUDGE NOT.

Think gently of the erring;
 Ye may not know the power
With which the dark temptation came
 In some unguarded hour.
Ye may not know how earnestly
 They struggled, or how well,
Until the hour of weakness came,
 And sadly thus they fell.

### Physiology for Nurses.

### NO. VII.—SPINAL COMPLAINTS.

In the last paper we talked a little about the spine, but there are still two or three more points to be noted about it before we dismiss it altogether. The bones which form it are made almost entirely of the spongy or cancellous tissue, and for that reason are specially liable to disease, which sometimes burrows a good distance into the bone before anyone, not even the owner of the spine, guesses what is going on. Another trouble to which the spine is liable is curvature, which usually appears first between the ages of twelve and eighteen, just when delicate girls are growing very fast, and probably out-growing their strength. It is very unfortunate that in these cases the spine does not keep pace with the other parts of the body—the head and chest grow, the shoulders broaden, but the strength of the spine does not increase fast enough to bear this extra weight, and so it bends. It is as if you were to try and balance a heavy stone on the end of a thin stick—the stick would bend, and the spine does just the same and for the same reason. There is no disease, it is merely weakness, and this trouble is usually laid upon girls; you get about nine cases of it in girls to one in boys. And another thing is, it is usually due to the occupations followed by girls, as distinct from those followed by boys, and besides, a boy will go and join some healthy game after his work is over.

That there need be little, if any, difference between most men and women, in the matter of muscular strength, is proved by what goes on amongst some barbarous tribes, where the men lead idle lives, and the women do all the work and carry the heavy weights, and their muscles grow very strong; and you certainly will not find spinal curvature amongst them—that is a trouble of civilised life. You must clearly understand that curvature of the spine is not a disease, nor due to disease, but is entirely the fault of a child's up-bringing, and her friends do her a grievous harm if they do not take measures in time to correct it, for in time it crushes the other organs in the body, and may shorten life. What ought to be attempted is: (1) to feed the child better; (2) to give her as much fresh air as you can possibly get for her; (3) not to let her sit up at a table or any work for many hours together without support to her back, but place her in a chair with a back, and prop her up well with pillows, so that the poor weak back does not get over-tired and yield; (4) if you can possibly avoid it, do not choose for a delicate over-grown girl work at which she must sit bolt upright, or in a stooping position; (5) whenever she can do so let her lie on a couch; (6) if she is short-sighted take her to the eye department and get her spectacles. A child with spinal mischief will have a sharp pain in the back if it jumps off ever so little a height, and if this happens once he will not be in a hurry to do it again, but you will notice that he moves about carefully, and will not hurry himself, and have the sprightliness of a healthy child. The position which a child will choose as the most comfortable in spinal disease should also warn an experienced eye—he will lean forward, and often grasp the thighs very firmly—a position very much like that which boys assume when playing leap-frog. Of course the child finds this position the least painful because, to some extent, it takes the weight of the body off the diseased spinal bones and throws it on the upper part of the legs. Another point to remember is that a child will often, when the disease has attacked the upper part of the spinal bones, complain of pain at the back of the head. Of course this may be merely a headache, but if it is repeated often this warning must not be overlooked. Again, if the disease is in the upper part of the spine, the child's neck may be stiff, so that when he wants to turn his head he will turn all his body round, and to look upwards he will bend the lower part of his back, and also a child will often make a habit of resting his chin on the top of the breast-bone, or of supporting it on the hand. Children with disease of the spine will lose flesh. I wish all district nurses would make these early signs of spinal disease entirely their own. It is such a terrible trouble to children, and at best and in the most hopeful cases it means months of weary lying on their backs. Some doctors say it is never cured if once it makes any way; others say it takes a year and a half or two years, not only of lying in bed, but of lying still on their backs as flat as possible. Do not let a child become constantly thinner without any apparent reason without asking the doctor about it. Half the battle in spinal disease is to recognise the evil and to treat it early, not to wait until it forces itself upon your notice in the form of a lump, which shows that the centre part of the bones of the spine are softening and melting away, and the rest of the spine bends at a sharp angle; and these bones may even break through completely, and the child die suddenly. The children of poor parents must go to a hospital where they get can the proper kind of splints to keep them perfectly quiet and prevent the diseased bones from rubbing each other. So much for the more serious of the two—actual disease of the spine. But to return once more to curvature. You will probably notice first of all that one of the shoulder-blades grows out more than the other, and, moreover, another point you notice is that this shoulder is higher than the other, one breast seems higher than the other, possibly one hip-bone sticks out more than the other. At the beginning of the trouble you will not usually find there is pain—that comes later on when the signs having been overlooked, the spine has become still more curved and presses on the internal organs. The owner of a curved spine lives a more and more miserable life and finally dies, her life having been ruined. What is needed for a crooked back is rest in the first place, and, finally, an apparatus to support the back and keep it straight.

### Everybody's Opinion.

*[Correspondence on all subjects is invited, but we cannot in any way be responsible for the opinions expressed by our correspondents. No communications can be entertained if the name and address of the correspondent is not given, or unless one side of the paper only be written on.]*

### PENSIONS.

S. C. N. writes: I met a nurse the other day who pays £1. 6s. quarterly to a pension fund organised by the hospital for which she works, but, however disagreeable her position may become, she can never leave that hospital without forfeiting all this money she puts by at such a great sacrifice. She is chained to that hospital for ever. Now, when nurses' pensions are really made a "national" affair, we shall be able in leaving one institution to join another, to have our pensions transferred; this is a great point many nurses do not seem to comprehend.

### EXAMINATION QUESTIONS.

A Country Nurse writes: The specimens of examination questions have interested me; I have been waiting for them to be answered, and then for them to supersede the puzzles. Will you not encourage nurses to answer them? Think of the hundreds of nurses in, for instance, country workhouses like this one—no resident doctor, no lectures, no library, no examinations, and little or no training (very often) to start with. Could they not teach themselves much by answering such questions as those you publish? They would have to be more observant of and interested in their patients, and they would have to read. But forgive me for saying that the questions are too numerous and too comprehensive. It would give too much additional work in a busy life. In order to make a beginning, I have answered the three first as well as I could, and I am sure many nurses would work at them if you gave prizes for the best answers.

CLEAN NAILS.

Student H. writes: I should like to know whether "Doris" wears her nails long or short. If she wears them long, and has to handle anything that is not particularly clean, I should say she would very soon get dirt collecting under them, which would not come off by washing in the ordinary way. Besides, it is very few people that have their hands continually in water. In my opinion nothing looks worse than dirty and badly-trimmed nails.

### Winter Amusements and Remunerative Work.

AN EXHIBITION OF DOLL NURSES.

The Editor of The Hospital has much pleasure in offering to nurses prizes to the value of £10 for the three best dressed sets of dolls representing the nursing staff of any hospital. The dolls must not be shorter than four inches nor taller than twelve inches, and must represent the sister, charge-nurse, and probationer of the hospital. Any nurse intending to compete must send her name and address on a post-card, headed "Doll Competition," together with the name of the institution or hospital, the uniform of which is to be faithfully reproduced, to the Editor of The Hospital, The Lodge, Porchester-square, London, W. *before September 1.* Further particulars will be announced later on, and arrangements will be made for a public exhibition of the dolls where nurses will be able to see the show. The number and amount of the prizes will not be fixed at present in deference to a general wish, and suggestions on this point are invited.

### Presentations.

On the occasion of Miss J. Mackay's leaving the Western Infirmary, Glasgow, she was presented, by her nurses, with a very handsome leather travelling bag. Miss Mackay has been trained at the Western, and for four years has held the post of sister in Dr. Finlayson's wards.

Mrs. Nicholls, late Sister Talbot of the London Hospital, has been presented by the resident medical staff with a handsome tea service, and by the sisters with a lamp, on the occasion of her marriage with Dr. Frederick Smith.

### Death in Our Ranks.

It is with great regret we record the death of Miss Hughes, at Cairo, from typhoid fever. Miss Hughes only went to Cairo about a year ago, on receiving the appointment of nursing sister at the Kaisr-el-Aini Hospital; and since she has been there she has won golden opinions amongst the European and native population. Miss Hughes trained at St. Bartholomew's, and then went to Netley. She was amongst the army sisters to volunteer for the Egyptian Campaign. The funeral took place last Sunday, and was largely attended by numerous friends.

We also regret to record the death of Nurse E. Sabel, at the London Hospital, from diphtheria. Nurse Sabel trained at the London, and had been engaged on private cases, from which she ever brought back excellent testimonials.

### Appointments.

[It is requested that successful candidates will send a copy of their applications and testimonials, with date of election, to The Editor, The Lodge, Porchester-square, W.]

Miss Sara Hotham, who was trained at the London Hospital, has been appointed to the post of district nurse at Esher, Surrey. The Duchess of Albany has always taken an interest in this institution, the arrangements of which are under H.R.H.'s patronage.

### Vacancies.

The following vacancies are announced:

**Matron.**

Swansea Hospital, £50.

**Nurses.**

Birmingham Fever Hospital (several).

Bootle-cum-Linacre Hospital, £25.

Bournemouth Home for Nurses (several).

Bradford Fever Hospital (two), £23.

Carlisle Infirmary (night), £24.

Cheltenham Institute.

Grantham Hospital.

Guisborough Miners' Accident Hospital, £30.

Hospital for Sick Children, E. (three), £25.

Jersey Institute, £25.

Kent Nursing Institution, West Malling (several).

Liverpool City Hospital, £30.

Liverpool Hospital, Park Hill (two), £25.

Lock Hospital, W.

Mrs. Rawlinson, Brighton.

Newcastle-on-Tyne, £25.

Regent-street, Nottingham, £25.

Rochdale Infirmary, £22.

Southport Infirmary, £25.

Southport Nurses' Home.

Stepney Hospital for Children.

St. Helens Hospital, £20.

St. Leonards-on-Sea.

Tunbridge Wells General Hospital, £25.

Victoria Home, Bournemouth.

West Bromwich Hospital, £22.

**District Nurses.**

Bowdon Nursing Establishment.
Woodsgate, Pembury, £50.

**Probationers.**

Canterbury Nurses' Institute.

Ventnor Consumption Hospital, £10.

Wirral Children's Hospital, Birkenhead.

### Notes and Queries.

To Correspondents.—1. Questions may be written on post-cards. 2. Advertisements in disguise are inadmissible. 3. In answering a query please quote the number. 4. If a private answer is desired, a stamped addressed envelope must be forwarded.

Notice.—We must really ask our correspondents to be more particular in enclosing name and address. Constantly we receive queries or letters with no name attached.

**Queries.**

(72) *Home Wanted.*—Can you tell me of a home where an aged person can be received? A small sum could be paid for her. *M.*

(73) *Books on Nursing.*—Please tell me what books to read to learn nursing and physiology? *Mary.*

(74) *Meat Jelly.*—Will any nurse give the receipt for making Anstey's meat jelly? *Private Nurse.*

(75) *How to Become a Nurse.*—I have a daughter who wishes to become a nurse. What should she do? *Pater.*

(76) *Home Wanted.*—A comfortable home for an incurable male patient wanted; either free, or very small payment. *A. E.*

**Answers.**

(67) *Private Nursing in States.*—Association of Trained Nurses, West Thirty-fourth-street, New York. The nurses pay 15 dollars down, and take their own fees; these vary from £2 to £4 a week.

(71) *Monthly Nurse.*—Cullingworth's "Manual of Monthly Nursing" is the best. *Vi.*

(71) "Monthly Nurse" may read any one of the following: Smith's "Handbook for Midwives," 3s. 9d.; Cullingworth's "Monthly Nursing," 1s. 3d.; Burton's "Handbook for Midwives," 4s. 6d. *J. C. H.*

(71) "Monthly Nurse" will find "The Handbook of Nursing," published by the Connecticut School of Nursing, Philadelphia, the best book to buy. Price, I think, 4s. 6d. It is a most useful book for all nurses, and is the one in use at Queen Charlotte's Lying-in Hospital. Churchill's "Manual for Midwives and Monthly Nurses" is also good, but "Monthly Nurse" must remember that no book will be of much use unless she goes into a hospital for training, so as to get theory and practice together. *E. A. Bedingfeld.*

(72) *Home Wanted.*—See answers to query (66) last week. Write to Nurse Williams, Maiden Newton, Dorchester.

(73) *Books on Nursing.*—Shepherd's "First Aid to the Injured," price 1s., teaches the elements of physiology and nursing at the same time. Domville's "Manual," price 2s. 6d., published by Churchill, is excellent, so is Luckes's "Lectures on Nursing," price 2s. 6d. "Physiology Primer," by Dr. Foster, price 1s., published by Macmillan, is clear and simple.

(75) *Pater* had better read the article on "How to Become a Nurse," which we publish this week. *Ed.*

*Grateful Patient.*—You cannot do better than present her with a surgical case, such as is advertised in our pages.

*Nurse Foster.*—Domville's "Manual for Hospital Nurses" can be had through any bookseller, or direct from the publishers, Messrs. Churchill, 11, New Burlington-street, London, W.

